# Smoking is a predictor of complications in all types of surgery: a machine learning-based big data study

**DOI:** 10.1093/bjsopen/zrad016

**Published:** 2023-04-22

**Authors:** Helene L Gräsbeck, Aleksi R P Reito, Heikki J Ekroos, Juhani A Aakko, Olivia Hölsä, Tuula M Vasankari

**Affiliations:** Department of Pulmonary Medicine, HUS Heart and Lung Center, Helsinki, Finland; Doctoral Programme of Clinical Research, University of Helsinki, Helsinki, Finland; Center for Musculoskeletal Diseases, Tampere University Hospital, Tampere, Finland; Faculty of Medicine and Health Technology, Tampere University, Tampere, Finland; Pulmonary Unit, HUS Porvoo Hospital, Porvoo, Finland; Medaffcon Oy, Espoo, Finland; Medaffcon Oy, Espoo, Finland; Department of Pulmonary Diseases and Clinical Allergology, University of Turku, Turku, Finland; Finnish Lung Health Association (Filha), Helsinki, Finland

## Abstract

**Background:**

Machine learning algorithms are promising tools for smoking status classification in big patient data sets. Smoking is a risk factor for postoperative complications in major surgery. Whether this applies to all surgery is unknown. The aims of this retrospective cohort study were to develop a machine learning algorithm for clinical record-based smoking status classification and to determine whether smoking and former smoking predict complications in all surgery types.

**Methods:**

All surgeries performed in a Finnish hospital district from 1 January 2015 to 31 December 2019 were analysed. Exclusion criteria were age below 16 years, unknown smoking status, and unknown ASA class. A machine learning algorithm was developed for smoking status classification. The primary outcome was 90-day overall postoperative complications in all surgeries. Secondary outcomes were 90-day overall complications in specialties with over 10 000 surgeries and critical complications in all surgeries.

**Results:**

The machine learning algorithm had precisions of 0.958 for current smokers, 0.974 for ex-smokers, and 0.95 for never-smokers. The sample included 158 638 surgeries. In adjusted logistic regression analyses, smokers had increased odds of overall complications (odds ratio 1.17; 95 per cent c.i. 1.14 to 1.20) and critical complications (odds ratio 1.21; 95 per cent c.i. 1.14 to 1.29). Corresponding odds ratios of ex-smokers were 1.09 (95 per cent c.i. 1.06 to 1.13) and 1.09 (95 per cent c.i. 1.02 to 1.17). Smokers had increased odds of overall complications in all specialties with over 10 000 surgeries. ASA class was the most important complication predictor.

**Conclusion:**

Machine learning algorithms are feasible for smoking status classification in big surgical data sets. Current and former smoking predict complications in all surgery types.

## Teaser

Machine learning algorithms are feasible for smoking status classification in large data sets. Current and former smoking associate with overall and critical complications in all types of inpatient and outpatient surgery of various invasiveness.

## Introduction

Globally, tobacco use causes more than 8 million deaths per year and is the leading preventable cause of death^1^. A recent American, state-wide study found that 24.1 per cent of surgical patients smoked cigarettes. This is higher than the national average in the general population^2^.

Smoking increases the risk of surgical complications through several mechanisms. It promotes atherosclerosis by altering the lipid profile, damaging the vascular endothelium, and increasing oxidative stress, neutrophil count, and hypercoagulability^3^. Further, cigarette smoke components impair wound healing, thereby increasing the risk of wound dehiscence and infection^4,5^.

In 2014, a comprehensive meta-analysis of 100 cohort and seven case–control studies found an elevated risk of general morbidity rate, intensive care admission, general infections, and pulmonary, wound, and neurological complications in smokers undergoing various types of surgery^6^. A narrative review conducted in 2015 found consistent evidence, stressing the risk of postoperative wound healing-related and cardiovascular complications in smokers^7^.

However, most studies on smoking-related postoperative complications are small and, of the cohort studies included in the 2014 meta-analysis, only two had a study population of over 100 000. Most of the studies only included one or few surgical specialties, and the large-volume specialty gynaecology was not covered in any of the studies^6^.

When studying large data sets, determining smoking status based on electronic health record (EHR) free-text notes often forms a major obstacle. The manual extraction of smoking status from EHRs is both laborious and expensive. Natural language processing (NLP) methods have proven feasible for clinical text classification tasks^8^. Furthermore, some recent studies have demonstrated that machine learning-based NLP methods are accurate in smoking status extraction from EHR notes^9–11^.

The aims of this study were to develop a well-functioning MLA for the classification of smoking status in a large patient population and to use this to assess smoking and former smoking as risk factors for postoperative complications in all types of inpatient and outpatient surgery, utilizing a large real-life sample. In addition, a further aim was to clarify the global importance of smoking as a risk factor for surgical complications in relation to other known risk factors.

## Methods

### Study sample and data source

This was a retrospective cohort study. The data comprised all surgical procedures performed between 1 January 2015 and 31 December 2019 in the Finnish hospital district of Helsingin ja Uudenmaan Sairaanhoitopiiri (HUS). HUS is a specialized healthcare provider and the largest hospital district in Finland, with a population base of roughly 1.7 million. The data source was HUS Datalake, a public database service for clinical researchers. HUS Datalake contains pseudonymized information from the local EHR systems, such as diagnosis codes, free-text clinical notes, and clinical measurement results. The patients were identified with the aid of Opera, an electronic notification created for each patient undergoing a procedure. Exclusion criteria were age below 16 years, non-surgical procedure, unknown smoking status, and unknown ASA class.

### Outcomes and explanatory variables

The primary outcome was the composite of any perioperative or postoperative 90-day complication in all surgeries. ICD-10 diagnosis codes for wound, cardiovascular, neurological, respiratory, thromboembolic, gastroenterological, urinary, and orthopaedic complications and unspecified bacterial infections, and the occurrence of death, reoperation, hospital readmission, and mechanical ventilation or ICU admission were included in the outcome composite (*Table S1*). Any of these events recorded within 90 days after surgery was considered a complication. Secondary outcomes were any 90-day complication in specialties with more than 10 000 operations (gastroenterological, orthopaedic, gynaecological, plastic, and otorhinolaryngological surgery), and, in all surgeries, critical 90-day grade IV–V (life-threatening complications and death) complications as defined in the Clavien–Dindo classification^12^. These critical complications were acute coronary syndrome, shock, cerebral infarction, pulmonary embolism, peritonitis, mechanical ventilation or ICU admission, and death. Severe complications of grade III (requiring re-intervention) were not included in this secondary analysis, as this was a registry study and determination of individual patient paths would not have been possible.

Demographic data included age and sex. Patient characteristics included preoperative smoking status, ASA class, and chronic diseases as classified in the Charlson co-morbidity index (CCI). CCI was calculated using ICD-10 codes, as implemented in the co-morbidity R package^13,14^.

### Definition of smoking status and training of the machine learning algorithm

EHR free-text sentences containing the word stems tupak* (‘tupakka’ meaning tobacco in Finnish and ‘tupakointi’ meaning smoking), aski* (‘aski’ meaning pack), and smok* were utilized for smoking status determination. For this purpose, all EHR free-text notes recorded by physicians between 2014 and 2019, including all medical specialties, were retrieved from HUS Datalake. A random sample of sentences were annotated manually into smoking status classes for training and testing of the MLA. Two medical doctors (H.L.G. and contributor J.N.) independently annotated the sentences according to predefined rules. According to West^15^, long-term smoking abstinence has often referred to abstinence for at least 6 months. Applying this definition, a current smoker had smoked any amount of cigarettes within the previous 6 months, an ex-smoker had stopped smoking at least 6 months previously, and a never-smoker had never smoked. If the smoking status could not be determined, it was annotated as unknown. Users of electronic cigarettes were assigned as unknown smoking status. The sentences were annotated in the HUS Datalake analysis environment.

The annotated sentences were converted into lower case and all special characters were removed. The sentences were split into training data (90 per cent of the annotated sentences) and test data (10 per cent of the annotated sentences). A supervised machine learning-based text classifier fastText^16^ was applied to classify smoking-related sentences by smoking status into the classes ex-smoker, never-smoker, current smoker, and unknown. An unknown class was used to filter unclear and uncertain sentences. Moreover, the classified sentences with low classification probabilities were assigned to the unknown class, where the thresholds for the classification probabilities were tuned in favour of specificity. MLA training and assessment of the MLA was performed using five-fold cross-validation.

Finally, surgery-level classification was performed by assigning a smoking status of ex-smoker, never-smoker, or current smoker to the patient if smoking-related sentences classified into one of those three classes were found in the medical record. If multiple smoking statuses were available for a patient prior to surgery and they were conflicting, the status of the most recent classification was assigned to the surgery, unless the status was a never-smoker, in which case the smoking status assigned to the surgery was an ex-smoker. Further details of the MLA training process and performance have been published elsewhere^17^.

### Statistical analysis

The impact of smoking status (never-smoker, ex-smoker, and current smoker) on postoperative complications was assessed using binary logistic regression unadjusted and adjusted with age as a continuous covariate and sex, CCI, and ASA class as categorical covariates. Surgeries with missing covariate values were omitted. Analyses were performed for all surgeries concerning overall complications and critical complications, and independently for specialties with more than 10 000 operations concerning overall complications. Corresponding unadjusted and adjusted ORs with 95 per cent c.i. were calculated. The relative importance of the variables in the model was assessed by calculating the partial chi-squared statistic for each variable, as well as a global importance score based on Shapley values^18^, which were calculated as implemented in the fastshap R package^19^. Nagelkerke’s pseudo R^2^ and McFadden’s pseudo R^2^ were computed to assess the overall model fit.

Data processing, statistical analysis, and modelling were performed using Python (version 3.8.4)^20^ and R (version 3.6.3)^21^. All relevant data are within the paper and its *Supplementary material*.

## Results

**Fig. 1:**
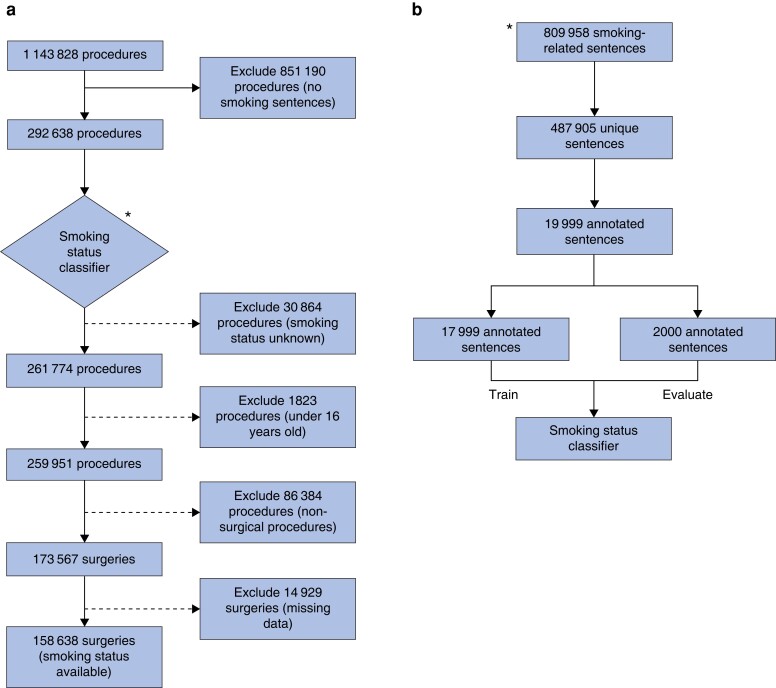
Sample selection and development of the machine learning algorithm **a** Study flow diagram displaying the total number of procedures in the retrieved data, the number of procedures excluded at each step of the selection process, and the number of surgeries included in the final sample. **b** A proportion of all extracted unique smoking-related sentences were annotated manually into smoking status classes for training and testing of the machine learning algorithm.

Roughly 500 000 unique sentences were retrieved from HUS Datalake, out of which 19 999 were randomly sampled and annotated manually into smoking status classes for training and testing of the MLA (*Fig. 1*). Initially, all sentences were classified into classes: ex-smoker, current smoker, never-smoker, and unknown. After tuning the threshold for uncertain classifications, the overall accuracy was 79.0 per cent (95 per cent c.i., 77.3 to 80.8 per cent) with precision of 95.8 per cent (95 per cent c.i., 93.5 to 98.1 per cent), 97.4 per cent (95 per cent c.i. 95.8 to 98.9 per cent), 95.0 per cent (95 per cent c.i. 93.5 to 96.6 per cent), and 55.8 per cent (95 per cent c.i. 51.5 to 60.1 per cent), and recalls of 68.7 per cent (95 per cent c.i. 63.4 to 73.9 per cent), 93.2 per cent (95 per cent c.i. 90.7 to 95.7 per cent), 67.6 per cent (95 per cent c.i. 64.4 to 70.9 per cent), and 92.0 per cent (95 per cent c.i. 89.6 to 94.3 per cent) for the ex-smoker, never-smoker, current smoker, and unknown classes respectively. When excluding the unknown class and assessing the performance of the MLA separately for the classes ex-smoker, never-smoker, and current smoker, the average precision and recall were 96.1 per cent and 84.6 per cent respectively.

**Fig. 2:**
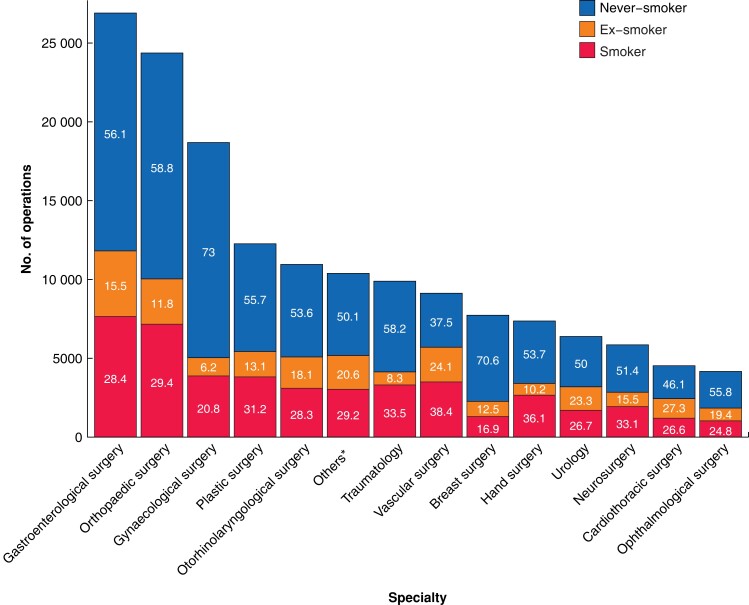
Surgical specialties: numbers of operations and smoking status class proportions Surgical specialties included in the final sample, with their numbers of operations, and numbers of never-smokers, ex-smokers, and current smokers. Percentages are shown within the boxes. *Includes thoracic surgery, endocrine surgery, general surgery, liver surgery, and transplant surgery.

**Fig. 3:**
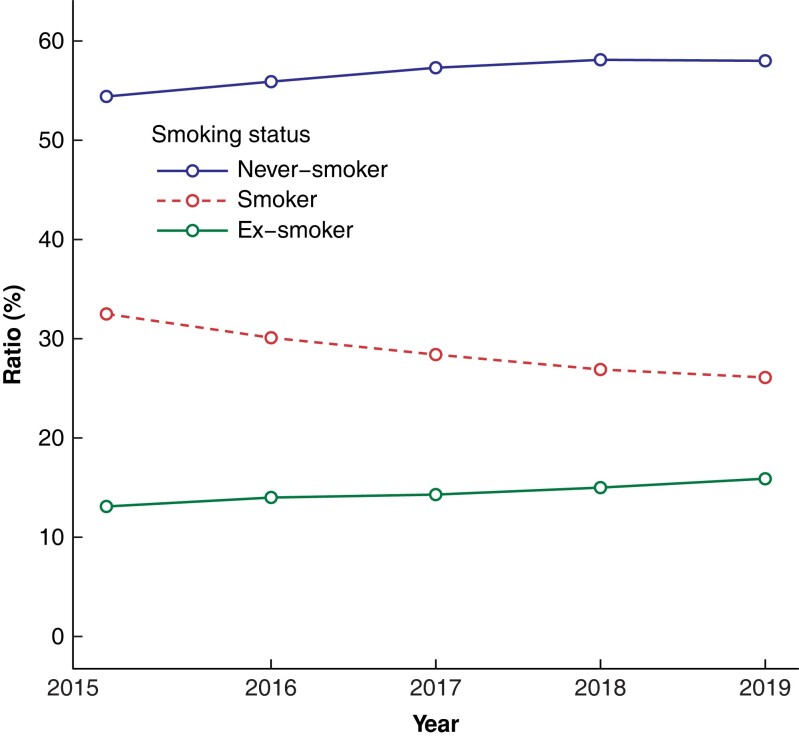
Surgical patients’ smoking status ratios in the study years 2015–2019

Smoking-related sentences were found in 292 638 of 1 143 828 procedures (*Fig. 1*). Smoking status was assigned to 261 774 procedures using the trained MLA. The final sample included 158 638 surgeries within 19 surgical specialties (*Fig. 2*), after removing non-surgical procedures, procedures with missing data, and patients under 16 years of age. The proportions of current smokers in the surgical specialties varied between 16.9 per cent in breast surgery (1308 of 7732 patients) and 38.4 per cent in vascular surgery (3503 of 9128 patients) (*Fig. 2*). The ratio of current smokers to the number of surgeries showed a downward trend during the study interval, from 33 per cent (6639 of the total of 20 435 surgeries) in 2015 compared with 26 per cent (9518 of the total of 36 488 surgeries) in 2019 (*Fig. 3*).

The patients’ baseline characteristics and demographics are displayed in *Table 1*. Ex-smokers were the oldest, with a median age of 66 (range 56–74) years, compared with 55 (range 41–65) years in current smokers and 57 (range 41–70) years in never-smokers. Ex-smokers were the most likely to be male and had the largest co-morbidity burden, with a large proportion in ASA class III or IV and having numerous diseases included in the CCI. They were also the most likely to have any of the potentially smoking-related CCI diseases.

**Table 1:** Baseline characteristics

Characteristic	Never-smoker, *n* = 90 208	Ex-smoker, *n* = 23 101	Current smoker, *n* = 45 326
**Sex**			
Male	32 199	13 586	23 290
Female	58 009	9515	22 036
**Age (years)**			
Mean(s.d.)	56(18)	64(14)	53(16)
Median (i.q.r.)	57 (41–70)	66 (56–74)	55 (41–65)
ASA class*			
1	19 656 (21.8)	1551 (6.7)	4977 (11.0)
2	37 924 (42.0)	6911 (29.9)	18 365 (40.5)
3	25 126 (27.9)	9 914 (42.9)	15 573 (34.4)
4	6964 (7.7)	4449 (19.3)	5828 (12.9)
5	538 (0.6)	276 (1.2)	583 (1.3)
CCI*			
0†	54 177 (60.1)	7860 (34.0)	24 734 (54.6)
1†	10 750 (11.9)	4328 (18.7)	7621 (16.8)
2†	16 595 (18.4)	5311 (23.0)	7007 (15.5)
3†	4064 (4.5)	2336 (10.1)	2851 (6.3)
4†	1638 (1.8)	1128 (4.9)	1265 (2.8)
≥5†	2984 (3.3)	2138 (9.3)	1848 (4.1)
**Acute myocardial infarction‡**	2058 (2.3)	1465 (6.3)	1602 (3.5)
**Congestive heart failure‡**	2468 (2.7)	1784 (7.7)	1374 (3.0)
**Peripheral vascular disease‡**	3944 (4.4)	3470 (15.0)	4859 (10.7)
**Cerebrovascular disease‡**	5640 (6.3)	2692 (11.7)	3571 (7.9)
**Chronic pulmonary disease‡**	4829 (5.4)	3742 (16.2)	4393 (9.7)
**Hemiplegia or paraplegia‡**	272 (0.3)	94 (0.4)	198 (0.4)
**Cancer (any malignancy)‡**	18 465 (20.5)	6892 (29.8)	7636 (16.8)
**Metastatic solid tumour‡**	1260 (1.4)	566 (2.5)	661 (1.5)
**Other co-morbidity**	5035 (5.6)	2401 (10.4)	3566 (7.9)

Values are *n* (%) unless otherwise indicated. *Percentages have been rounded and might not total 100. †Total number of CCI co-morbidities. ‡Potentially smoking-related CCI co-morbidities. CCI, Charlson co-morbidity index.

The overall complication rate was 56 690 (35.7 per cent) in all surgeries. Corresponding figures were 9921 of 26 903 (36.9 per cent) in gastroenterological surgery, 6364 of 24 372 (26.1 per cent) in orthopaedic surgery, 4982 of 18 691 (26.7 per cent) in gynaecological surgery, 5523 of 12 265 (45.0 per cent) in plastic surgery, and 2440 of 10 955 (22.3 per cent) in otorhinolaryngological surgery. The critical complication rate was 6925 (4.4 per cent) in all surgeries. Logistic regression analysis of all surgeries showed that both ex-smokers and current smokers had increased odds of overall postoperative complications compared with never-smokers (*Table 2*). The same was true after multivariable adjustment, with OR 1.09 (95 per cent c.i. 1.06 to 1.13) for ex-smokers and OR 1.17 (95 per cent c.i. 1.14 to 1.20) for current smokers. Both ex-smokers and current smokers had increased unadjusted overall complication odds in all secondary analyses. After adjustment for covariates, odds were significantly increased for ex-smokers in gastroenterological surgery (OR 1.09; 95 per cent c.i. 1.01 to 1.18), plastic surgery (OR 1.24; 95 per cent c.i. 1.10 to 1.40), and otorhinolaryngological surgery (OR 1.20; 95 per cent c.i. 1.05 to 1.37). For current smokers, odds were significantly increased in all specialties (OR 1.21 (95 per cent c.i. 1.13 to 1.29) in gastroenterological surgery, OR 1.36 (95 per cent c.i. 1.27 to 1.46) in orthopaedic surgery, OR 1.12 (95 per cent c.i. 1.03 to 1.21) in gynaecological surgery, OR 1.20 (95 per cent c.i. 1.09 to 1.31) in plastic surgery, and OR 1.16 (95 per cent c.i. 1.03 to 1.30) in otorhinolaryngological surgery). The unadjusted odds of critical complications in all surgeries were increased for both ex-smokers and current smokers, and this was also true after adjustment, with OR 1.09 (95 per cent c.i. 1.02 to 1.17) for ex-smokers and OR 1.21 (95 per cent c.i. 1.14 to 1.29) for current smokers.

**Table 2:** Complication rates and association between smoking status and outcomes

Outcome	Specialty	Smoking status		
		Never-smoker	Ex-smoker	Current smoker
Any 90-day complication	All surgeries	27 994 (31.0)	10 401 (45.0)	18 295 (40.4)
	Unadjusted	1 (Reference)	1.82 (1.77 to 1.87)*	1.5 (1.47 to 1.54)*
	Adjusted	1 (Reference)	1.09 (1.06 to 1.13)*	1.17 (1.14 to 1.20)*
	Gastro	4902 (32.5)	1847 (44.4)	3172 (41.4)
	Unadjusted	1 (Reference)	1.66 (1.55 to 1.78)*	1.47 (1.39 to 1.56)*
	Adjusted	1 (Reference)	1.09 (1.01 to 1.18)*	1.21 (1.13 to 1.29)*
	Ortho	3328 (23.2)	893 (31.1)	2143 (29.9)
	Unadjusted	1 (Reference)	1.49 (1.37 to 1.63)*	1.41 (1.32 to 1.5)*
	Adjusted	1 (Reference)	1.01 (0.92 to 1.11)*	1.36 (1.27 to 1.46)*
	GYN	3477 (25.5)	357 (30.9)	1148 (29.6)
	Unadjusted	1 (Reference)	1.31 (1.15 to 1.49)*	1.23 (1.14 to 1.33)*
	Adjusted	1 (Reference)	1.12 (0.98 to 1.28)*	1.12 (1.03–1.21)*
	Plastics	2647 (38.7)	868 (54.0)	2008 (52.5)
	Unadjusted	1 (Reference)	1.86 (1.67 to 2.08)*	1.75 (1.62 to 1.90)*
	Adjusted	1 (Reference)	1.24 (1.10 to 1.40)*	1.20 (1.09 to 1.31)*
	ORL	1065 (18.1)	584 (29.5)	791 (25.5)
	Unadjusted	1 (Reference)	1.88 (1.68 to 2.12)*	1.55 (1.39 to 1.72)*
	Adjusted	1 (Reference)	1.20 (1.05 to 1.37)*	1.16 (1.03 to 1.30)*
Critical 90-day complication	All surgeries	2974 (3.3)	1577 (6.8)	2374 (5.2)
	Unadjusted	1 (Reference)	2.15 (2.02 to 2.29)*	1.62 (1.53 to 1.71)*
	Adjusted	1 (Reference)	1.09 (1.02 to 1.17)*	1.21 (1.14 to 1.29)*

Values are *n* (%) unless otherwise indicated. *Odds ratio (95% c.i.). Gastro, gastroenterological surgery; Ortho, orthopaedic surgery; GYN, gynaecological surgery; Plastics, plastic surgery; ORL, otorhinolaryngological surgery.

When assessing relative variable importance, ASA class was the most important complication predictor in a majority of the models (*Table 3*). Concerning overall complications, smoking status had mean Shapley values (absolute log odds scale) of 0.07 in all surgeries, 0.08 in gastroenterological surgery, 0.13 in orthopaedic surgery, 0.04 in gynaecological surgery, 0.10 in plastic surgery, and 0.08 in otorhinolaryngological surgery. The corresponding Wald (X) % values were 1.1, 1.5, 7.5, 1.3, 2.1, and 1.5 respectively. In the critical complication model, smoking status had a mean Shapley value of 0.09 and a Wald (X) % value of 0.70. The overall complication model including all surgeries had a McFadden’s pseudo R^2^ of 0.12 and a Nagelkerke’s pseudo R^2^ of 0.20.

**Table 3:** Relative variable importance

Outcome	Specialty	Statistic	Variable						
			SS	Age (years)	Sex (male)	ASA	CCI	M*P*R^2^	NPR^2^
Any 90-day complication	All surgeries	Wald X	141.4	209.3	584.4	10 437.4	1425.2	0.12	0.20
		Wald X (%)	1.1	1.6	4.6	81.6	11.1		
		Shapley	0.07	0.12	0.14	0.79	0.24		
	Gastro	Wald X	34.2	4.9	15.2	1820.7	370.6	0.13	0.21
		Wald X (%)	1.5	0.20	0.70	81.1	16.5		
		Shapley	0.08	0.04	0.05	0.78	0.28		
	Ortho	Wald X	76.8	10.8	88.6	734.0	112.4	0.07	0.12
		Wald X (%)	7.5	1.1	8.7	71.8	11.0		
		Shapley	0.13	0.07	0.15	0.52	0.12		
	GYN	Wald X	9.0	92.0	17.1	462.6	102.8	0.04	0.06
		Wald X (%)	1.3	13.5	2.5	67.7	15.0		
		Shapley	0.04	0.21	0.01	0.38	0.13		
	Plastics	Wald X	21.1	19.5	171.9	753.6	31.3	0.12	0.20
		Wald X (%)	2.1	2.0	17.2	75.6	3.1		
		Shapley	0.10	0.12	0.26	0.78	0.12		
	ORL	Wald X	10.1	0.60	26.8	153.5	498.8	0.11	0.17
		Wald X (%)	1.5	0.10	3.9	22.3	72.3		
		Shapley	0.08	0.03	0.13	0.31	0.47		
Critical 90-day complication	All surgeries	Wald X	39.1	29.6	15.3	5386.1	538.4	0.19	0.22
		Wald X (%)	0.70	0.50	0.30	89.6	9.0		
		Shapley	0.09	0.11	0.05	1.2	0.29		

Gastro, gastroenterological surgery; Ortho, orthopaedic surgery; GYN, gynaecological surgery; Plastics, plastic surgery; ORL, otorhinolaryngological surgery; SS, smoking status; CCI, Charlson co-morbidity index; M*P*R^2^, McFadden’s pseudo R^2^; NPR^2^, Nagelkerke’s pseudo R^2^.

## Discussion

In this large database study, an MLA for smoking status classification based on EHR notes was successfully developed. Both former and current smoking significantly increased the risk of overall 90-day postoperative complications in all surgeries. Ex-smokers had a significantly increased overall 90-day complication risk in gastroenterological, plastic, and otorhinolaryngological surgery. For current smokers, this risk was increased in all secondary analysis specialties (gastroenterological, orthopaedic, gynaecological, plastic, and otorhinolaryngological surgery). The risk of 90-day critical complications in all types of surgery was increased in both former and current smokers.

Compared with the Michigan study, the current population had a somewhat larger proportion of current smokers (25 *versus* 29 per cent)^2^. In the general Finnish population in 2017, 13 per cent of men and 10 per cent of women aged 20 to 84 years were daily smokers^22^. It must be stressed that a large group of patients with unknown smoking status were excluded from the analyses, and the true proportion of current smokers in the sample may differ from this study’s results. However, based on both this study and previous ones, the smoking problem among surgical patients is apparent. The smoking trend in this study was downward. This may reflect changes in attitudes towards smoking and effects of increased tobacco taxation.

EHR free-text notes are often heterogeneous and inexact, and distinguishing between current and ex-smokers as per this study’s definition often proved impossible and made the classification task complex. Examples of sentences classified into the unknown category are ‘has smoked before’, ‘has smoked x pack-years’, ‘long smoking history’, and ‘stopped smoking recently’. Sentences such as ‘stopped smoking 3/20’, ‘stopped smoking in the 60s’, and ‘smoked 1995–2011’ were considered accurate enough to be classified into the ex-smoker category.

To reliably assess smoking status as a predictor of surgical complications, this study aimed to maximize the reliability of smoking status classifications at the expense of a higher number of samples for statistical analysis. In other words, the high proportion of true positives (precision) for the ex-smoker, current smoker, and never-smoker classes was emphasized, while achieving a high recall for the unknown sentences. Therefore, sentences with unknown smoking status were also included in the training of the MLA and thresholds were set for the probabilities that were required for a sentence to be classified as an ex-smoker, current smoker, or never-smoker. Because of this and the strict criteria set for classifying ex-smokers, direct comparison of this study’s results with those of other studies utilizing MLA methods for smoking status classification is difficult.

In their Finnish study on the effect of smoking status on cancer patient mortality rate, Karlsson *et al*.^23^ also used MLA methods for smoking status classification. Algorithms used were BERT and ULMFiT, which require more computing power than fastText. The BERT model performed the best, with an average precision of 88.2 per cent for the classes ex-smoker, smoker, and never-smoker, compared with the current study precision of 96.1 per cent. Karlsson *et al*.^23^ reported performance metrics at the patient level while performing a performance assessment at the sentence level consisting of only unique sentences.

Hawn *et al*.^24^ were the first ones to investigate smoking-related postoperative complications after major surgery in a large study population. Similar to this study, the main finding was increased rates of complications in both current and ex-smokers compared with never-smokers in adjusted analyses. In contrast, the authors reported complication subgroups and stratified the population by pack-year, finding an exposure greater than 20 pack-years to significantly increase the complication risk. However, non-cardiac and emergency cases were excluded, and multivariable analyses did not adjust for sex or co-morbidities.

Turan *et al*.^25^ chose to propensity-match current smokers with never-smoker controls, utilizing data on major surgeries from a national quality improvement programme. Current smokers had an increased likelihood of 30-day mortality rate and major complications, which is consistent with the results of this study. In addition, a linear relationship between the odds of any major morbidity rate and the amount of smoking was found in a smaller subset of matched current smokers. In contrast, an ex-smoker group was not included, and patients with severe preoperative disease were excluded.

In a comprehensive study in 2013, Musallam *et al*.^26^ investigated the effects of current and former smoking on postoperative mortality rate and vascular and respiratory events after major surgery. Only current smokers had increased odds of mortality rate, whereas both current and ex-smokers had increased odds of arterial and respiratory events. The odds of arterial and respiratory events increased with pack-year exposure.

Schmid *et al*.^27^ investigated the effects of smoking on postoperative complications in 16 major cardiovascular, orthopaedic, and oncological surgical procedures. Current smokers had increased odds of overall, pulmonary, wound, and septic complications after most cardiovascular and oncological procedures compared with never-smokers. Ex-smokers also had increased odds of such complications, but to a lesser extent.

In orthopaedic surgery, smoking-related problems include non-union of bones in fracture surgery^28^, surgical site infection in spine surgery^29^, and higher mortality rate and analgesia usage in hip and knee arthroplasty^30^. In gastroenterological surgery, smoking has been found to increase anastomotic leakage after colorectal surgery^31–33^, infections during pancreatic resection^34^, and the risk of incisional hernia after laparotomy^35^. In gynaecological surgery, the risks of smoking have not been studied extensively. In observational study settings, smoking has been found to increase the risk of incisional hernia after open gynaecological surgery^36^ and severe complications after ovarian cancer surgery^37^. In plastic surgery, smoking has been recognized as a risk factor for wound complications, including infections, seroma, flap haematoma, flap necrosis, and dehiscence^38^. In otorhinolaryngological surgery, both former and current smoking increase the risk of wound complications after tumour resection involving total laryngectomy^39^. In otological procedures, smoking increases the risk of wound infections, wound dehiscence, and hospital readmission^40^. Complication subgroups were not analysed in this study, but the increased odds of overall complications in current smokers undergoing procedures of the aforementioned specialties are in line with the literature.

Based on the Shapley value-based global importance scores of this study’s relative variable importance models, smoking status is associated with postoperative complications to roughly the same extent as age, with some variation between the models. The strongest association between smoking status and complications was found in orthopaedic surgery. These findings were the same when calculating partial chi-squared statistics for the variables. This is noteworthy considering that smoking was the only influenceable variable in the models.

Ex-smokers were defined as having had quit smoking at least 6 months previously, and current smokers had higher odds of complications than never-smokers. However, the initiation of smoking cessation as early as 6 months before surgery is not realistic in most cases. This raises questions about the optimal timing and practices of cessation. Studies have shown that a preoperative smoking cessation interval of at least 4 weeks reduces complication rates. Each additional week increases the magnitude of effect by 19 per cent^41^. Short-term smoking cessation of less than 4 weeks does not seem to increase or lower the risks compared with current smoking^42,43^. However, no evidence indicates that patients cannot be advised to quit smoking at any time before surgery^43^. When it comes to smoking cessation support, evidence shows that intensive interventions should be initiated at least 4 weeks before surgery. The combination of behavioural support and pharmacotherapy has the largest effect on complication risk reduction^44^.

This study has some important strengths. First, the sample was large, and included a wide range of both inpatient and outpatient surgical procedures of variable invasiveness, providing valuable information on the effects of smoking in all types of surgery. The findings also demonstrate smoking-associated risks in gynaecological surgery, a sparsely studied topic. Second, a 90-day postoperative follow-up interval enables capturing of both early and late postoperative complications compared with a 30-day interval. Third, the utilization of an MLA was a novel approach to smoking status classification, and the results show that it is feasible in a population with EHRs in Finnish. Considering the challenge of distinguishing between current and ex-smokers, the predictive model performed well and required comparatively little computational power.

The study also has limitations. The data lacked information on pack-years and the number of cigarettes smoked, and the study findings rely on self-reported smoking status. The MLA did not consider tobacco products other than cigarettes. Due to the 6-month limit and the sometimes inexact EHR notes, the distinction between current smokers and ex-smokers sometimes proved challenging. Aiming for a classification as exact as possible led to the exclusion of a large group of patients with unknown smoking status from the analyses, which may have caused selection bias. In addition, the results of the MLA are not directly applicable to other languages than Finnish. With these limitations in mind, the study findings should be interpreted with some degree of caution. The inadequate smoking status recording in the EHRs in this study is certainly an important finding. At present, smoking status is not recorded in a systematic, structural, and extractable way in the HUS area. A future quality register could be a way to overcome this issue.

Based on the results of this study, MLAs appear feasible for smoking status classification in large data sets. Current and former smoking associate with complications in all types of inpatient and outpatient surgery of various invasiveness, and also with critical complications. The results suggest that the risks of surgical complications associated with smoking and increasing age are approximately equal. However, of these two, smoking is the only modifiable one. In the future, intervention studies on efficient preoperative smoking cessation models are needed in the surgical field as a whole, and in orthopaedic surgery in particular.

## Supplementary Material

zrad016_Supplementary_Data

## Data Availability

The data sets generated and analysed during the study are not publicly available, but are available from the corresponding author on request.
