# Supplementary material for: Smoking is a predictor of complications in all types of surgery: a machine learning-based big data study
Source: BJS Open. 2023 Apr 22;7(2):zrad016. doi: 10.1093/bjsopen/zrad016 (PMC10122503; doi:10.1093/bjsopen/zrad016)
Supplement: zrad016_Supplementary_Data [file zrad016_supplementary_data.docx]

**Smoking is a predictor of complications in all types of surgery: machine learning-based big data study**

Authors: Helene L Gräsbeck, MD, PhD student^1,2^, Aleksi R P Reito, MD, PhD, Assoc. Prof.^3,4^, Heikki J Ekroos, MD, PhD, Chief Physician^5^, Juhani A Aaakko, D.Sc. (Tech.), Data Scientist^6^, Olivia Hölsä, M.Sc. (Tech.), Jr. Data Scientist^6^, Tuula M Vasankari, MD, PhD, Professor^7,8^

^1^ Department of Pulmonary Medicine, HUS Heart and Lung Center, Finland

^2^ Doctoral Programme of Clinical Research, University of Helsinki, Finland

^3^ Center for Musculoskeletal Diseases, Tampere University Hospital, Finland

^4^ Faculty of Medicine and Health Technology, Tampere University, Finland

^5^ Pulmonary Unit, Porvoo Hospital, HUS, Finland

^6^ Medaffcon Oy, Espoo, Finland

^7^ University of Turku, Department of Pulmonary Diseases and Clinical Allergology, Finland

^8^ Finnish Lung Health Association (Filha)

**Corresponding author.** Helene Gräsbeck, Department of Pulmonary Medicine, HUS Heart and Lung Center, P.O. Box 340, 00029 HUS, Finland. helene.grasbeck@hus.fi **ORCID ID** 0000-0002-6297-8165

**Supplementary Materials - Index**

| **Supplementary Figures and Tables** |  |
| --- | --- |
| Table S1. | *pag. 2* |
| **References** | *pag. 3* |
|  |  |

| **Table S1.**  Composite outcome of postoperative complications. | |
| --- | --- |
| **Complication type** | **ICD-10 diagnosis code** |
| **Wound complications** |  |
| Wound infection | T81.4; T87.4; L03; L08.8; L08.9; A46; H62.0*A46; O86.0; N61; L02.2 |
| Hematoma | T81.0; T87.6; O69.5; O71.7; O90.2; I31.2; I62.0; I62.1; I69.2 N83.6; N83.7; P10.0; R58 |
| Wound dehiscence | T81.3; O90.0; O90.1 |
| **Cardiovascular complications** |  |
| Acute coronary syndrome**^*^** | I20-I24 |
| Hypotension | I95; O26.; R03.1 |
| Atrial fibrillation | I48 |
| Cardiac insufficiency | I50; I09.9; I11.0; I13.0; I13.2; I97.1; O29.1; O74.2; O75.4; O89.1 |
| Shock**^*^** | R57; T81.1; T88.2#; O75.1; O08.3 |
| **Neurological complications** |  |
| Cerebral infarction**^*^** | I63; I69.3; G46* |
| Neural lesion | S14; S24; S54; S64; S74; S84; S94; T06.2; T14.4; G54 |
| **Respiratory complications** |  |
| Pneumonia | J13; J14; J15; J16; J17*, J18; J69.0; J69.8; J85; O29.0; O74.0; O89.0 |
| Exacerbation of chronic obstructive pulmonary disease | J44.0, J44.1 |
| **Thromboembolic complications** |  |
| Pulmonary embolism**^*^** | I26; O08.2; O88.2 |
| Deep vein thrombosis | I80.1#; I80.20#; I80.29#; I80.3#; I80.8#; O22.3; O87.1 |
| **Gastroenterological complications** |  |
| Abdominal pain | R10; K30 |
| Ileus | K56; K31.5; K91.3 |
| Melena and gastrointestinal bleeding | K92 |
| Peritonitis**^*^** | K65; N73.3; N73.5 |
| Bile duct rupture | K83.2 |
| **Urinary complications** |  |
| Hematuria | R31; N02 |
| Urinary retention | R33; N13; N31 |
| Urinary tract infection | N10; N12; N13.6; N30; N39.0; N39.8; N39.9; O23; O86.2; O86.3; R82.7 |
| Kidney insufficiency | N17-N19; N99.0; I12.0; I13.1; I13.2; O08.4; O90.4 |
| Urinary bladder rupture | N32.4 |
| Urinary incontinence | R32; N39.3-4 |
| **Orthopaedic complications** |  |
| Prosthesis infection | T84.5 |
| Mechanical complication of fixation material | T84.1; T84.2 |
| Nonunion | M84.0; M84.1; M84.2 |
| Mechanical complication of endoprosthesis | T84.0 |
| Mechanical complication of other implant | T84.3; T84.4 |
| Fracture caused by prosthesis insertion | M96.6 |
| **Unspecified bacterial infection** | A49.9; O86.4 |
| **Death^*^** |  |
| **Reoperation** |  |
| **Hospital readmission** |  |
| **Mechanical ventilation or intensive care unit admission^*^** | WX730; WX892 |
| **^*^**Complications of Clavien-Dindo^1^ grade IV-V included in secondary analysis. | |

**References**

1. Dindo D, Demartines N, Clavien PA. Classification of surgical complications: A new proposal with evaluation in a cohort of 6336 patients and results of a survey. *Ann Surg*. 2004;240(2):205-213.
